# Trends and Determinants of Attitudes Towards People Living with HIV/AIDS Among Women of Reproductive Age in Tajikistan

**DOI:** 10.5195/cajgh.2019.349

**Published:** 2019-06-10

**Authors:** Hakim Zainiddinov

**Affiliations:** 1 Rutgers University, United States

**Keywords:** Tajikistan, HIV/AIDS, discriminatory attitudes, women, PLWHA

## Abstract

**Introduction:**

Despite having one of lowest rates of newly diagnosed HIV infections among former Soviet countries, Tajikistan has a substantial level of discriminatory attitudes towards people living with HIV/AIDS (PLWHA). While initial attempts were made to explore discriminatory attitudes of a wide range of professionals, women’s general attitudes towards PLWHA received less scholarly attention. Employing a nationally representative sample from the 2000 and 2005 Multiple Indicator Cluster Surveys (MICS), sociodemographic determinants of HIV-related discriminatory attitudes of women aged 15–49 in Tajikistan were identified and examined over time.

**Methods:**

A representative sample included 5,453 women of reproductive age from the capital city and four regions of Tajikistan. Two dichotomized scenarios representing the agreement to let an HIV-infected teacher continue teaching in school and the willingness to buy food from an HIV-infected cashier were constructed. Univariate and multivariable analyses of HIV-related discriminatory attitudes were obtained using Stata 14.

**Results:**

Insignificant but positive changes were observed in the women’s attitudes between 2000 and 2005. Logistic regression models showed that negative attitudes were associated with the lack of knowledge of HIV/AIDS prevention methods, endorsement of HIV/AIDS transmission misconceptions, and never having been tested for HIV (p≤0.001). Women living in the rural areas, married, with lower education, and from low income households were less tolerant towards PLWHA.

**Conclusions:**

The data from Tajikistan underscore the persistence of HIV-related discriminatory attitudes among low socioeconomic status women. The study findings can be potentially used to target the disadvantaged groups and guide the design and implementation of programs that promote voluntary HIV-testing, raise awareness about HIV/AIDS prevention methods, and help dispel transmission misconceptions.

## Introduction

Despite the recent advances in HIV prevention and treatment technologies that transformed HIV into a manageable disease, HIV-related stigma and discrimination still represent a problem. Tajikistan, reporting one of lowest rates of newly diagnosed HIV infections among the former Soviet countries,^1^ is not exception from this general trend. According to the first national study on discrimination and stigmatization of HIV-infected people conducted by the Strategic Research Center under the President of Tajikistan, nearly every third person (28.9%) held negative attitudes towards people living with HIV/AIDS (PLWHA).^2^ Discriminatory attitudes towards PLWHA have been reported in the form of labor and education restrictions, in the context of medical care, and within families and communities.^2^ Although initial attempts were made to explore discriminatory attitudes of a wide range of professionals, including medical personnel, law-enforcement men, service sector workers, teachers, local officials, mass media employees, lawyers, judges, and religious leaders,^2^ studies that examine women’s attitudes towards PLWHA in Tajikistan are nearly absent. Most of the previously published studies on HIV/AIDS in Tajikistan focused on high risk of HIV infection among injection drug users and labor migrants^3^ or assessed comprehensive HIV/AIDS knowledge among women.^3^,^4^ Consequently, a limited number of qualitative research looked at the discrimination against HIV-positive injection drug users by health care providers^5^, as well as labor migrants’ wives’ knowledge, attitudes, and behaviors about HIV/AIDS risk and protection.^6^ The aim of this study was to identify sociodemographic determinants of HIV-related discriminatory attitudes of a nationally representative sample of women aged 15–49 in Tajikistan and examine these changes over time.

## Methods

### Data source

The data were obtained from the 2000 and 2005 Multiple Indicator Cluster Surveys (MICS), conducted by the State Committee on Statistics of the Republic of Tajikistan with the financial and technical support of the United Nations Children’s Fund.^7^,^8^ The MICS employed a representative sample of women from the capital city and all four regions of Tajikistan. The final sample included 5,453 women aged 15–49 years. Both MICS surveys had high response rates of 98.8% and 96% for 2000 and 2005 respectively.^7^,^8^

### Measures

To measure discriminatory attitudes towards people living with HIV/AIDS, the 2000 survey included two questions: (1) “Should teacher with HIV/AIDS be allowed to teach in school?”; and (2) “Would you buy food from cashier with HIV/AIDS?”. The 2005 survey assessed discrimination using four questions: (1) “If a female teacher has the AIDS virus but is not sick, should she be allowed to continue teaching in school?”; (2) “Would you buy fresh vegetables from a shopkeeper or vendor if you knew that this person had the AIDS virus?”; (3) “If a member of your family became infected with the AIDS virus, would you want it to remain a secret?”; and (4) “If a member of your family became sick with the aids virus, would you be willing to care for him or her in your household?”. To be consistent across two years of the study, the first two questions from each survey were used.

Key independent variables were women’s knowledge about HIV/AIDS prevention methods, transmission misconceptions, and their HIV/AIDS testing status. Research has already reported the association between these predictors and discriminatory attitudes towards PLWHA.^9^,^10^,^11^,^12^,^13^,^14^ Following prior research,^3^ the first two independent variables were operationalized as followed. A score measure of knowledge about HIV/AIDS prevention methods was created by combining answers from three questions asking whether women knew that they can avoid the AIDS virus by (1) having one uninfected and faithful partner; (2) using condoms; and (3) not having sex at all. The second independent variable, which is also a score measure, was based on three questions: can people get infected with the AIDS virus through (1) witchcraft or other supernatural means, (2) from mosquito bites, and (3) whether or not it is possible for a healthy-looking person to have the AIDS virus. All three answers were combined and coded into a dichotomous variable signifying respondent who did not endorse myths and misconceptions about HIV/AIDS transmission. The study also employed a number of demographic and socio-economic status characteristics known to affect discriminatory attitudes towards HIV-infected people.^12^,^14^,^15^,^16^ Demographic characteristics were captured through variables such as age (ranging from 15 to 49 years old), marital status (currently married versus all else), region of residence (a series of five dummy variables: Dushanbe, Khatlon, Sogd, Direct Rule Districts (DRDs) and Gorno-Badakhshan Autonomous Region (GBAO)), and area of residence (rural versus urban). Socioeconomic status measures were education (higher versus all else), and household income (recoded as tertiles: low, medium and high). The study used a dichotomous variable ‘year 2005’ that contrasted respondents who participated in the 2005 survey with those who did not.

### Statistical analysis

First, a univariate analysis was used to show changes across the analyzed years. Second, chi-square and t-tests were conducted to assess whether independent and control variables differ significantly on two attitudes. Finally, three sets of binary logistic regression models were run to identify main sociodemographic determinants of HIV-related discriminatory attitudes among women aged 15–49 years in Tajikistan. Model 1 displayed the logistic regression predicting tolerant attitudes towards PLWHA by three key independent measures: knowledge of HIV/AIDS prevention, myths about HIV/AIDS transmission, and having ever had an HIV/AIDS test. Model 2 added demographic characteristics. Model 3 built upon the previous model while controlling for socioeconomic characteristics. Stata 14 was used for all analyses.

## Results

**Figure 1:**
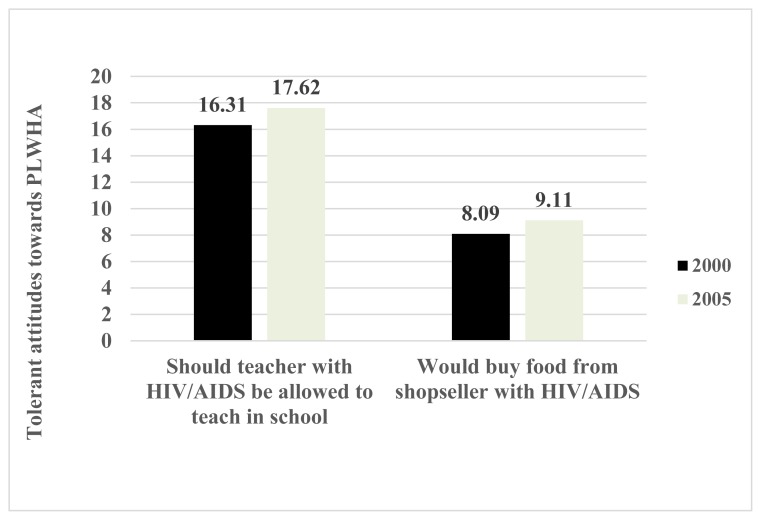
Trends in positive attitudes towards people living with HIV/AIDS

Figure 1 presents the change in attitudes towards PLWHA from 2000 to 2005. The percentages of women who did not express discriminatory attitudes have increased insignificantly for both questions/scenarios beteween 2000 and 2005. The change was slightly higher for respondents who believed that an HIV-positive teacher should continue teaching (1.31%) than for respondents who would not buy food from an HIV-positive cashier (1.02%).

Table 1 presents descriptive statistics for all variables used in the study. Overall, less than one in five (17.88%) respondents believed that an HIV-positive teacher should be allowed to teach. Only one in ten (9.02%) said that they would buy food from an HIV-positive cashier.

Significant differences in tolerance towards PLWHA were observed on all measures, except for the age variable on allowing an HIV-positive teacher to teach in school. Significantly higher proportions of respondents who knew about HIV/AIDS prevention and transmission methods and had ever been tested for HIV/AIDS said that an HIV-positive teacher should continue teaching and that they would buy food from an HIV-positive cashier. Compared to respondents from other regions of the country, significantly higher proportions of respondents from the capital city of Dushanbe and GBAO had positive attitudes to HIV-infected teachers and cashiers. Significantly higher proportions of unmarried women and those who live in urban areas expressed less discriminatory attitudes compared to their married and rural dweller comparisons. For example, the percentage of urban respondents who believed that an HIV-positive teacher should continue teaching was twice as high (24.65%) in comparison to those from rural areas (12.32%). Additionally, the proportion of women who showed less HIV discriminatory attitudes had higher socioeconomic status characteristics.

Table 2 reports results from the three sets of binary logistic regression models. As the baseline models (columns 1 on both panels) indicated, all three key independent measures were strong predictors of stigmatizing attitudes, except for the insignificant association between knowledge of HIV/AIDS prevention and buying food from an HIV positive cashier. Women who had knowledge on HIV/AIDS prevention methods, correctly identified myths about HIV/AIDS transmission, and had ever been tested for HIV/AIDS, were respectively 1.63, 4.45, and 1.46 (p≤0.001) times more likely than their counterparts to believe that an HIV-positive teacher should continue teaching. Similarly, women who did not endorse myths and had an HIV/AIDS test before, were over eight (OR=8.36, p≤0.001) and near two times (OR=1.74, p≤0.001) more likely to buy food from an HIV-positive cashier.

No substantial changes occurred once demographic and socioeconomic status characteristics were introduced in Models 2 and 3. Overall, the effects of three key predictors of stigmatization attitudes persisted in these models (columns 2 and 3 on both panels). A noticeable difference was observed with respondents who had an HIV/AIDS test. The magnitudes of effects for these respondents who believe that an HIV-positive teacher should continue teaching attenuated by 10% and further 5% with the introduction of demographic and socioeconomic measures respectively, turning the previously highly significant estimate (OR=1.46, p≤0.001) into significant (OR=1.31, p≤0.05) and marginally significant (OR=1.24, p≤0.10) when comparing the coefficients in Model 1 to Model 2 and Model 2 to Model 3. The introduction of the demographic characteristics increased the magnitudes of effects nearly by 5%, turning the highly significant estimate into significant (OR=1.74, p≤0.001 vs OR=1.82, p≤0.05), whereas the inclusion of socioeconomic controls decreased the magnitudes and returned the estimate to highly significant (OR=1.82, p≤0.05 vs OR=1.76, p≤0.001).

There was no association between age and discriminatory attitudes. Marital status and rural location were negatively associated, whereas education and household income were positively associated with discriminatory attitudes. On the first outcome (an HIV-positive teacher should continue teaching in school), women living in the capital city of Dushanbe were less likely to report discriminatory attitudes than respondents from all regions of the country, except for GBAO. Similar associations were observed on the second outcome (buying food from an HIV-positive shopseller), where women from Khatlon joined women from GBAO in reporting less discriminatory attitudes than women from Dushanbe.

## Discussion

Using a nationally representative sample, the study aimed to identify sociodemographic determinants of HIV-related discriminatory attitudes of women aged 15–49 in Tajikistan, as well as to examine changes over time. Over five years, changes in women’s attitudes occurred insignificantly or at very low rates. The shortage of HIV/AIDS awereness program and incentives for voluntary HIV testging, due to the slow recovery of the country following a five-year civil war from 1992–1997, might account for such low and insignificant changes. The key finding of the study, demonstrating that women’s knowledge of HIV prevention and transmission methods and HIV testing are significant predictors of tolerant attitudes towards PLWHA, supports this assertion. The the association remained significant even after controlling for demographic and socioeconomic status characteristics. Prior research indicates that during the period of 2000–2005 there was a two-fold increase in general knowledge about HIV/AIDS, accompanied by a substantial decrease in the ability to correctly identify prevention methods and transmission misconceptions among women of reproductive age in Tajikistan.^3^ With the availability of next waves of the survey, the study should be replicated to continue monitoring changing trends of discriminatory attitudes towards PLWHA. Some positive changes were shown by recent studies. According to the 2007 National Study, the percentages of people who believed that an HIV-infected teacher should be allowed to teach and that they would buy food from an HIV-positive vendor were 39.4% and 23.3% respectively.^2^

The findings on the first two main predictors are consistent with numerous previous studies.^12^,^13^,^14^ Concerning the third predictor, the finding corroborates with some studies that found negative association between the probability of being tested and HIV stigmatizing attitudes^10^,^11^,^12^ and contradicts others that demonstrated that HIV testing was not a significant factor in achieving positive attitudes towards PLWHA.^13^ Our findings suggests the importance of HIV testing in reducing discriminatory attitudes. Efforts should be made towards programs that promote and encourage voluntary HIV testing. Debilitating fear of encountering discrimination affects negatively women’s intentions, whereas health education on HIV counselling and testing increases their willingness to test for HIV.^17^

Consistent with previous research, it was found that women’s tolerant attitudes were positively associated with education, high income, and urban residency.^12^,^16^ Women in poverty, less educated, and from rural areas were found to be more likely to express discriminatory attitudes towards PLWHA.

With the exception of women from GBAO, respondents from other regions reported higher rates of discriminatory attitudes than those from the capital. This could be linked to high rates of education among the GBAO women. These women participate more actively in household decisions, have higher rates of modern contraceptives use, highest median age at first marriage, and more advanced median age at first birth,^18^ which are comparable to women with higher education.

The finding on significance of marital status is consistent with previous research.^15^ Married women were more likely to report discriminatory attitudes. An explanation can be sought in fear related to AIDS.^15^ Tajikistan has an estimated one million male labor migrants who are “likely to have unprotected contacts with sex workers and return home without HIV testing.”^6^ Fear concerning contracting the disease might shape negative attitudes of married women whose husbands construct the backbone of labor migrant force of the country.

The study has several limitations. First, the MICS survey focuses on women’s attitudes towards PLWHA, as this study has not collected data from people experiencing discriminatory acts. Prior research demonstrates a weak relationship between attitudes and discrimination.^19^,^20^ Future surveys should include questions that measure PLWHA’s own perceptions of HIV-related discrimination. A significantly negative impact of perceived HIV stigma on quality of PLWHA’s both physical and psychosocial dimensions of life has been demonstrated by previous research.^21^ Second, since the data focus primarily on female respondents, it is not possible to identify gender differences in attitudes towards people living with HIV/AIDS. Prior studies show that men express discriminatory attitudes towards HIV-infected people at higher rates than women.^12^ Third, the data do not reveal socioeconomic status of PLWHA. Studies demonstrate that discriminatory attitudes are more austere towards marginalized groups, such as homosexual men, sex workers, and injection drug users who engaged in behaviors that potentially led to the disease.^9^ Finally, since the current MICS surveys on Tajikistan were conducted 14 and 19 year ago, applicability of such data to current situation can raise questions. With the emergence of new data, the study should be replicated to demonstrate evolution of attitudes over the current period.

Although worldwide advances in medicine, technology, and treatment have transformed HIV into a managed and chronic disease, pervasiveness and harmful consequences of HIV-related discrimination remain acute. Combatting negative attitudes towards PLWHA to ensure that PLWHA enjoy their human rights, maintain a good quality of life, and continue to be productive local and global citizens is an essential component of the global HIV response toward the creation of an AIDS-free generation. While local communities, national governments, and international organizations around the globe employ policies and implement programs to reduce HIV-related discrimination, empirical studies identifying effective interventions to guide such policies are crucial. The data from Tajikistan underscore the persistence of HIV-related discriminatory attitudes among women of reproductive age. Consistent with previous research, lack of adequate knowledge on HIV/AIDS prevention and transmission methods and restraining practices on HIV testing are shown to be powerful factors that shape negatively women’s attitudes towards PLWHA. The study suggests the need for programs that promote voluntary HIV-testing, raise awareness about HIV/AIDS prevention methods, and help dispelling transmission misconceptions aimed at reduction of HIV-related discrimination. These findings also call for designing and implementing comprehensive interventions for women who are in poverty, married, live in rural areas, and less educated to change their attitudes towards PLWHA. Given the importance of reducing HIV-related discrimination worldwide, the findings from Tajikistan could inform policy interventions in the former Soviet Central Asian countries with similar sociopolitical background and economic transformation, as well as in other countries across the world with low HIV prevalence.

## Figures and Tables

**Table 1:** Proportion of respondents showing positive attitudes, MICS 2000, 2005

Characteristics	Should teacher with HIV/AIDS be allowed to teach in school	Would buy food from shopseller with HIV/AIDS
**Knowledge of HIV/AIDS prevention**	***	**
Yes	19.79%	9.58%
No	11.58%	7.17%
**Myths about HIV/AIDS transmission**	***	***
Do not endorse myths	19.66%	10.07%
Endorse myths	4.64%	1.24%
**Ever had HIV/AIDS test**	***	***
Yes	25.14%	14.23%
No	17.08%	8.45%
**Age**		
In years (range: 15–49)	30.21 9.69	29.64 9.73**
**Marital status**	***	***
Currently married	14.96%	6.73%
Not married	23.66%	13.55%
**Region of residence**	***	***
Dushanbe	27.84%	11.88%
Khatlon	13.60%	9.07%
Sogd	11.84%	4.98%
DRD	8.09%	3.18%
GBAO	23.07%	14.58%
**Residence type**	***	***
Urban	24.65%	11.76%
Rural	12.32%	6.78%
**Education**	***	***
Else	13.80%	7.37%
Higher	33.78%	15.45%
**Household income**	***	***
Low	9.32%	5.75%
Medium	13.35%	6.65%
High	24.97%	12.14%
N	**5,453**	**5,453**
Percent	**17.88%**	**9.02%**

Notes: Means and standard deviations are presented for continuous variables; proportions (%) are presented for categorical variables. Proportions are given for answer “yes.”

t-tests are used to assess significant differences between means for continuous variables; χ^2^ are used for categorical variables

* p ≤ .05,

** p ≤ .01,

*** p ≤ .001.

**Table 2:** Binary logistic regression predicting positive attitudes towards people living with HIV/AIDS, MICS 2000, 2005

	Should teacher with HIV/AIDS be allowed to teach in school			Would buy food from shopseller with HIV/AIDS		

	Model 1	Model 2	Model 3	Model 1	Model 2	Model 3
Knowledge of HIV/AIDS prevention	1.63*** (1.34–1.97)	1.61*** (1.32–1.98)	1.51*** (1.23–1.85)	1.15 (0.91–1.47)	1.23 (0.95–1.58)	1.17 (0.91–1.52)
Myths about HIV/AIDS transmission	4.45*** (3.05–6.47)	3.45*** (2.36–5.05)	3.04*** (2.07–4.45)	8.36*** (4.13–16.94)	5.87*** (2.89–11.95)	5.39*** (2.65–10.98)
HIV/AIDS test (yes)	1.46*** (1.16–1.83)	1.31* (1.04–1.67)	1.24+ (.98–1.58)	1.74*** (1.31–2.32)	1.82* (1.35–2.46)	1.76*** (1.30–2.38)
Age (range: 15–49)		1.01 (0.95–1.07)	.98 (0.92–1.04)		1.01 (0.94–1.09)	0.99 (0.91–1.07)
Currently married (ref. Not married)		0.60*** (0.50–0.72)	0.66*** (0.54–0.79)		0.49*** (0.39–0.63)	0.53*** (0.41–0.67)
Khatlon (ref. Dushanbe)		0.64*** (0.49–0.84)	0.79+ (0.59–1.04)		1.20*** (0.86–1.69)	1.40+ (0.99–1.98)
Sogd (ref. Dushanbe)		0.60*** (0.48–0.76)	0.69** (0.54–0.87)		0.69* (0.50–0.94)	0.75* (0.54–1.04)
DRD (ref. Dushanbe)		0.44*** (0.32–0.61)	0.50*** (0.36–0.70)		0.49** (0.30–0.80)	0.54* (0.33–0.89)
GBAO (ref. Dushanbe)		1.43** (1.13–1.82)	1.47** (1.15–1.88)		2.25*** (1.66–3.06)	2.35*** (1.73–3.19)
Rural (ref. Urban)		0.54*** (0.44–0.65)	0.74** (0.59–0.92)		0.55*** (0.43–0.71)	0.71* (0.54–0.95)
Higher education (ref. Other)			2.05*** (1.73–2.43)			1.52*** (1.22–1.91)
Medium income (ref. Low income)			1.14 (0.89–1.46)			0.87 (0.63–1.19)
High income household (ref. Low income)			1.55*** (1.19–2.02)			1.30 (0.93–1.82)
Year 2005	1.08 (0.88–1.31)	0.85 (0.69–1.05)	0.99 (0.80–1.23)	1.20 (0.92–1.57)	0.87 (0.66–1.16)	0.96 (0.72–1.28)
	0.03***	0.09***	0.08***	0.01***	0.02***	0.02***

Notes: Effect estimates are presented as odds ratios. Confidence intervals are given in parentheses.

+ p < 0.10,

* p ≤ 0.05,

** p ≤ 0.01,

*** p ≤ 0.001.
